# Perception of occupational therapy intervention in wheelchair seating among healthcare professionals in Bahrain

**DOI:** 10.3389/fresc.2026.1797993

**Published:** 2026-04-10

**Authors:** Yathreb Aladraj, Mohamed Altajer, Ahmed Almarhoon, Hassan Izzeddin Sarsak

**Affiliations:** 1 Department of Rehabilitation and Physiotherapy, Salmaniya Medical Complex, Government Hospitals, Salmaniya, Bahrain; 2 Allied Health Services, Eastern Health Cluster, Dammam, Saudi Arabia; 3 Occupational Therapy Program, Batterjee Medical College, Jeddah, Saudi Arabia

**Keywords:** Bahrain, interprofessional collaboration, occupational therapy, referral patterns, rehabilitation, wheelchair seating

## Abstract

**Background:**

Proper wheelchair seating is essential for functional independence and quality of life. However, the role of Occupational Therapy (OT) in these interventions is often undervalued within multidisciplinary teams, particularly in regions like Bahrain where specific systemic barriers are not fully understood.

**Objective:**

To investigate the perceptions, awareness, and referral practices of Bahraini healthcare professionals (HCPs) regarding OT in wheelchair seating and mobility (WSM).

**Methods:**

A descriptive cross-sectional survey was conducted among 110 licensed HCPs in Bahrain, primarily physical therapists (34.5%), nurses (27.3%), and medical doctors (18.2%). Data were collected on professional awareness, self-rated knowledge, referral history, and systemic barriers. Statistical analyses included Pearson's Chi-Square, Kruskal–Wallis H tests, and Binary Logistic Regression to identify predictors of clinical referrals.

**Results:**

While 80.9% of respondents were aware of the OT role, only 33.6% had ever referred a patient to OT for seating, revealing a profound “knowledge-practice gap”. OT interventions were highly regarded, with 74.5% of participants viewing them as “very effective”. Primary obstacles to referral included a lack of HCP awareness (78.2%), limited OT availability (71.8%), and high equipment costs (63.6%). Binary logistic regression identified formal training as the only significant independent predictor of referral behavior (*p* = 0.005); those without training had 82% lower odds of initiating a referral. No significant correlation was found between years of professional experience and self-rated knowledge (*p* = 0.887).

**Conclusion:**

Despite a high level of professional regard for OT, systemic barriers and a critical lack of specialized training hinder its clinical integration in Bahrain. To optimize wheelchair service delivery, healthcare institutions must prioritize structured interprofessional education (IPE) and implement standardized digital referral pathways within electronic medical records.

## Introduction

Wheelchair seating and positioning are foundational components of rehabilitation, directly influencing comfort, mobility, posture, energy expenditure, and overall participation in daily activities (1). Proper seating supports functional independence, reduces secondary complications, and enhances quality of life for individuals who rely on wheeled mobility (2, 3). Despite this centrality, the role of occupational therapy (OT) in wheelchair seating is frequently undervalued within multidisciplinary teams, which can contribute to delays in intervention and suboptimal outcomes for wheelchair users (4).

A systematic, client-centered approach to seating is essential to optimize outcomes, yet practice patterns vary across settings and regions, highlighting a need for greater clarity on OT's contribution to seating interventions (3, 4). Global guidelines, such as those proposed by the World Health Organization (WHO) for wheelchair service delivery, advocate four sequential steps to standardize service provision. These steps emphasize interprofessional collaboration, patient empowerment, and continuity of care (1, 4). Within this interprofessional framework, physical therapists predominantly address restoring mobility and functional independence, while occupational therapists focus on adapting activities of daily living (ADLs) to accommodate seating needs, promoting self-management, postural control, and pressure-relief strategies. Although both professions share overlapping competencies, their complementary expertise yields the most comprehensive seating interventions (5, 6).

The rationale for this study stems from the observation that while global guidelines advocate for a structured approach, translating these principles into routine practice remains uneven, and evidence supporting a single, unified approach to wheelchair seating assessment and provision is limited (6–8). In Bahrain and similar contexts, gaps persist in knowledge transfer, referral pathways, and access to OT-led seating services. Therefore, the primary purpose of this study is to elucidate how Bahraini healthcare professionals perceive OT involvement in wheelchair seating, identify specific barriers to referral and collaboration, and inform targeted educational and policy initiatives to optimize seating services. By mapping current attitudes against actual referral patterns, the research seeks to bridge the identified “knowledge–practice gap” and provide a framework for strengthening interprofessional collaboration and evidence-based interventions for wheelchair users in the region.

## Methods

### Study design and recruitment

This study employed a descriptive cross-sectional survey design to characterize the perceptions of healthcare professionals (HCPs) regarding occupational therapy (OT) interventions in wheelchair seating and mobility (WSM) in Bahrain. Participants were recruited over a specific period using a combination of convenience and purposive sampling. Recruitment channels included institutional email lists, professional networks, hospital assemblies, and professional associations. To address the recruitment denominator, 155 HCPs were directly invited to participate via targeted outreach. A total of 110 completed responses were collected, yielding an estimated response rate of 71%. This response rate ensures that the findings are representative of the cohort actively engaged in these professional networks.

### Participants and sample justification

The final sample of 110 participants comprised a multidisciplinary cohort including Physical Therapists (34.5%), Nurses (27.3%), Medical Doctors (18.2%), and Occupational Therapists (10.9%). The inclusion of Occupational Therapists (OTs) was intended to establish a baseline for professional awareness and to evaluate whether OTs themselves perceive systemic barriers to the services they provide. The sample size was determined based on a precision rationale to provide stable descriptive statistics and sufficient power for inferential analysis. Specifically, the sample was large enough to perform Binary Logistic Regression, which identified formal training as a significant predictor of referral behavior (*p* = 0.005) with an odds ratio of 0.18 for those without training.

### Representativeness and setting

The study targeted licensed HCPs across hospitals, rehabilitation centers, and private clinics. The cohort is highly experienced, with 63.6% of respondents having over 10 years of professional experience, reflecting a senior segment of the Bahraini healthcare workforce. While the sample is predominantly hospital-based (67.3%), it aligns with the primary setting where specialized wheelchair seating and mobility decisions are made in the Bahraini health system.

### Instrumentation

The research utilized a descriptive cross-sectional survey of 110 licensed HCPs in Bahrain. Data were collected using a structured, self-administered questionnaire that was developed and subsequently piloted to ensure feasibility and content validity. The tool primarily utilized closed-ended items, including Likert-type and categorical questions, to facilitate quantitative analysis. The instrument comprised several key sections that include: (1) demographics; professional background, setting, and years of experience, (2) knowledge and awareness; items assessing awareness of the OT role (Q4) and self-rated knowledge levels (Q5), (3) clinical practice; referral history (Q6) and perceived importance/effectiveness of OT (Q7–Q8), (4) perceived benefits; assessment of impact on comfort, pressure ulcer prevention, mobility, and postural support, and (5) barriers and training; identification of systemic challenges (Q10, Q15), adequacy of current interprofessional collaboration (Q11), and formal training history/needs (Q12–Q14) (see Supplementary Appendix A).

### Instrument development and validation

The survey instrument was developed through a systematic process to ensure it accurately captured the perceptions and clinical practices of healthcare professionals (HCPs) in Bahrain.

#### Item development

Items were generated based on the World Health Organization (WHO) Wheelchair Service Training Package (WSTP) and global literature regarding interprofessional collaboration in rehabilitation. The questionnaire focused on five domains: demographics, knowledge, clinical practice, perceived benefits, and systemic barriers.

#### Content validity

The instrument underwent expert review by senior rehabilitation specialists to ensure the technical accuracy of the terminology and the relevance of the clinical scenarios presented.

#### Pilot testing

A pilot study was conducted to evaluate the feasibility, clarity, and time-to-completion. This ensured that the self-administered tool was easily understood across diverse professional backgrounds, including nursing and medicine. The pilot group consisted of a subset (*n* = 28) of the target population (HCPs in Bahrain) to refine the tool.

#### Internal consistency and reliability

The tool demonstrated high sensitivity in detecting the “knowledge–practice gap,” as evidenced by the statistically significant *p* = 0.005 identifying formal training as a predictor of referral. Ordinal and Likert-type scales (e.g., perceived importance and effectiveness) showed distinct median values across disciplines, indicating the tool's ability to discriminate between varying professional attitudes (Median = 4.0 for OTs/PTs vs. Median = 3.0 for Nurses).

#### Minimization of bias

To strengthen internal validity, mandatory response checks were used to prevent missing data, and anonymity was strictly maintained to mitigate social desirability bias regarding clinical knowledge.

### Data collection procedures

The survey was primarily administered via an online platform, with paper-based versions available at selected sites to maximize participation. Participants provided consent before accessing the questionnaire. Mandatory response checks were implemented for key items to minimize missing data. Anonymity and confidentiality were strictly upheld to reduce social desirability bias.

### Statistical analysis

Data were analyzed using descriptive and inferential statistics to characterize the sample and examine professional associations. Descriptive statistics, including frequencies and percentages, were used to summarize participant demographics, awareness levels, and referral patterns. To examine the associations between professional profiles and clinical practices, the following inferential tests were employed: (1) Pearson's Chi-Square and Fisher's Exact Tests used to determine significant associations between categorical variables, specifically comparing professional discipline (PT, Nurse, MD, etc.) and work setting against referral history (Q6) and role awareness (Q4). Fisher's Exact Test was utilized where expected cell counts were less than five, (2) Spearman's Rank Correlation employed to assess the relationship between ordinal variables, such as years of experience and self-rated knowledge levels (Q5), (3) Kruskal–Wallis H Test used to compare differences in the perceived importance (Q7) and effectiveness (Q8) of OT interventions across different professional disciplines and experience groups, and (4) Binary Logistic Regression conducted to identify key predictors of clinical referrals, evaluating how factors like formal training (Q12) and professional discipline influence the likelihood of a healthcare professional initiating a referral to OT. The Binary Logistic Regression served as a *post-hoc* validation that the items effectively measured the intended construct (clinical referral behavior). For significant results, *post-hoc* pairwise comparisons were performed using the Dunn–Bonferroni correction to control for Type I error inflation associated with multiple comparisons. Effect sizes for these non-parametric comparisons were calculated using epsilon-squared, where values of 0.01, 0.08, and 0.26 represent small, medium, and large effects, respectively. All analyses were conducted with a significance level set at *p* < 0.05 to ensure statistical rigor in identifying the “knowledge–practice gap”.

### Ethical considerations

The study protocol received approval from the Research Committee for Government Hospitals in Bahrain (approval number 69-100725). Participation was entirely voluntary, and respondents retained the right to withdraw at any time. All data were stored securely and are reported only in aggregate form to protect participant identity.

## Results

### Sample overview and participant profile

The study achieved a total of 110 completed responses. The respondent pool was characterized by a high degree of professional experience and was primarily hospital-based. The participant pool was primarily hospital-based (67.3%) and highly experienced, with over 63% having more than 10 years in the field. The largest group of respondents was Physical Therapists (34.5%, *n* = 38), followed by Nurses (27.3%, *n* = 30) and Medical Doctors (18.2%, *n* = 20). Occupational Therapists comprised 10.9% (*n* = 12) of the sample. A substantial majority of the participants (63.6%, *n* = 70) reported having more than 10 years of professional experience. Most respondents were employed in hospitals (67.3%, *n* = 74), with a smaller portion working in rehabilitation centers (13.6%, *n* = 15) and private clinics (8.2%, *n* = 9) (see Table 1).

**Table 1:** Demographics and professional profile of study participants (*N* = 110).

Variable	Category	Frequency (*n*)	Percentage (%)
Professional Discipline	Physical Therapist	38	34.5%
	Nurse	30	27.3%
	Medical Doctor	20	18.2%
	Occupational Therapist	12	10.9%
	Allied Healthcare	9	8.2%
	Other	1	0.9%
Years of Experience	<5 Years	18	16.4%
	6–10 Years	22	20.0%
	>10 Years	70	63.6%
Work Setting	Hospital	74	67.3%
	Rehabilitation Center	15	13.6%
	Private Clinic	9	8.2%
	Other (e.g., Primary Care)	12	10.9%
Formal Training in WSM	Received Training	28	25.5%
	No Formal Training	82	74.5%

### Awareness and self-rated knowledge

A significant disparity was noted between general awareness of the occupational therapist's role and the depth of professional knowledge. While 80.9% (*n* = 89) of HCPs were aware of the OT role in wheelchair seating, nearly one-fifth (19.1%, *n* = 21) remained unaware. Despite high awareness, 68.2% of respondents rated their personal knowledge as either “not knowledgeable” (27.3%) or only “somewhat knowledgeable” (41.8%). Only 20.9% (*n* = 23) felt they were “very knowledgeable”. Role awareness was found to be universal (100%) among the Occupational Therapy and Physical Therapy subgroups. In contrast, awareness levels were significantly lower among other disciplines including medical doctors, nurses, and social workers at 26.3% (*p* < 0.001). Despite the inherent awareness within the OT subgroup, the overall sample still exhibited a profound knowledge–practice gap, as the high awareness of OTs and PTs did not statistically correlate with an increase in actual referral history (*p* = 0.604).

### Clinical referral patterns and knowledge levels

There is a profound “knowledge-practice gap” where high perceived value does not translate into clinical action. While 80.9% of HCPs (95% CI: 73.5%–88.3%) were aware of the OT role in wheelchair seating, only 33.6% (95% CI: 24.7%–42.5%) had actually referred a patient to OT for this service. Furthermore, only 22.7% (95% CI: 14.8%–30.6%) of participants felt that current interprofessional collaboration was adequate.

### Perceived benefits and challenges

**Figure 1:**
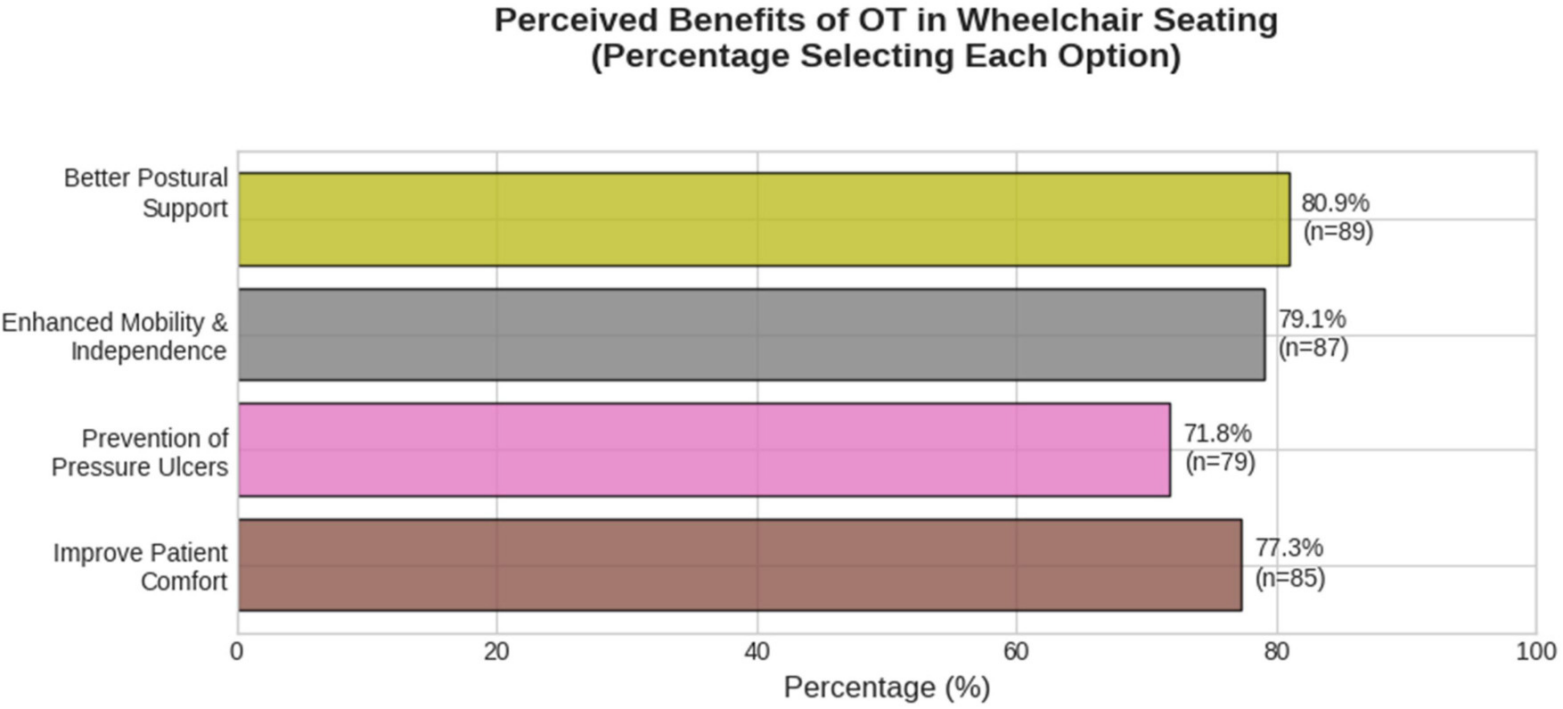
Perceived benefits of occupational therapy in wheelchair seating.

The most recognized clinical benefits of OT seating interventions were enhanced mobility/independence and better postural support (81.8% each). Prevention of pressure ulcers (74.5%) and improved patient comfort (73.6%) were also highly endorsed (see Figure 1). Respondents identified limited awareness among HCPs as the top challenge (78.2%). Other major challenges included a lack of resources (73.6%), environmental issues (73.6%), and limited training or expertise (68.2%).

### Collaboration and professional training needs

Only 22.7% (*n* = 25) of participants felt that current interprofessional collaboration regarding wheelchair seating was adequate. A critical deficit in specialized education was observed, with 74.5% (95% CI: 66.3%–82.7%) of practitioners lacking formal training in wheelchair seating. Professionals with formal training were significantly more likely to initiate referrals (56.0%) compared to those without training (24.7%). Spearman's Rank Correlation indicated no significant relationship between years of experience and self-rated knowledge (rs = −0.0137, *p* = 0.887), suggesting that clinical exposure alone does not bridge the knowledge gap. Among the small group that had received training, only 53.6% (*n* = 15) reported that the OT role was included in that training. There is a clear mandate for professional development, with 79.1% (*n* = 87) of HCPs expressing a desire for more workshops and training sessions.

### Systemic barriers in Bahrain

**Figure 2:**
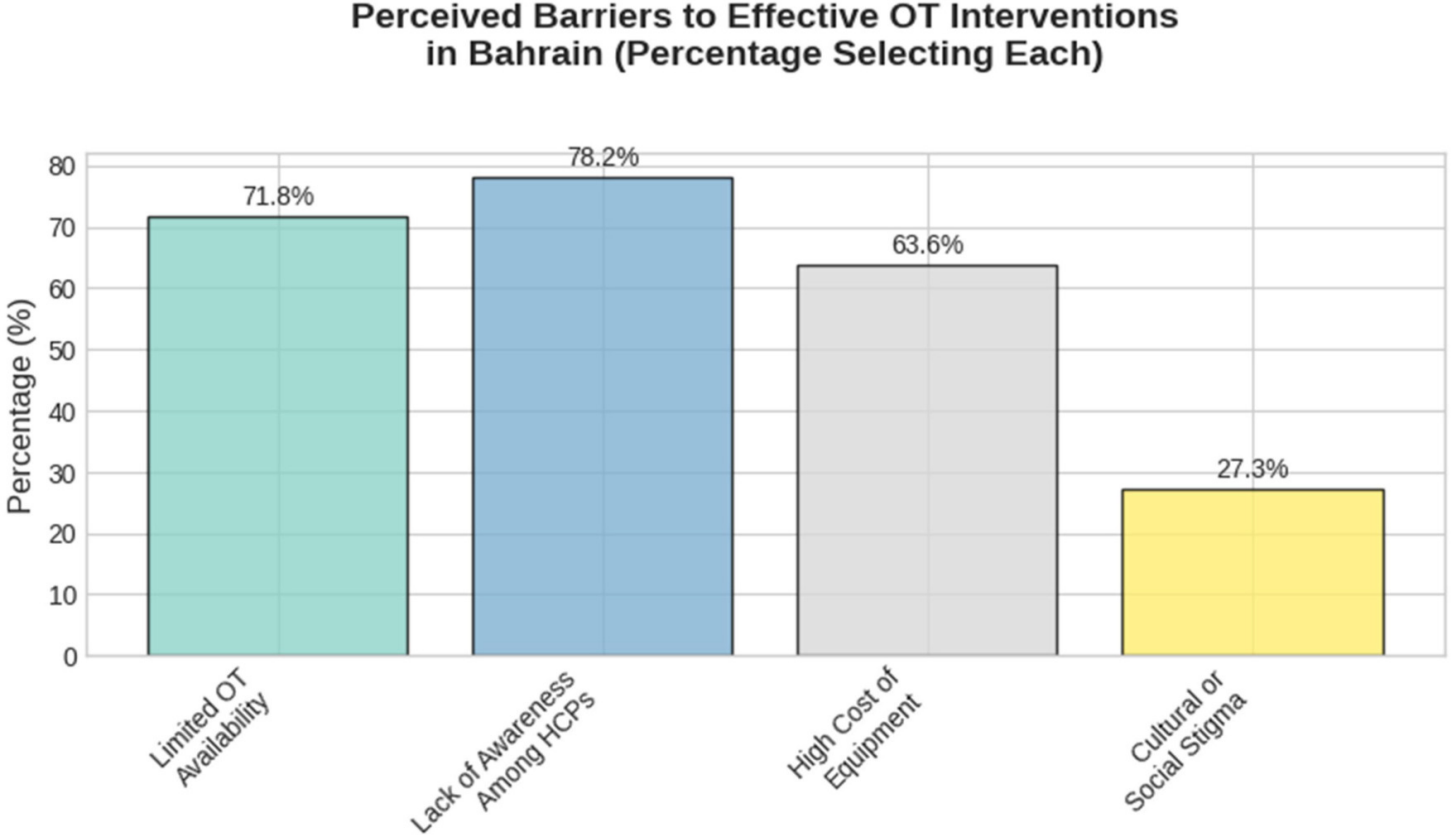
Top perceived barriers to effective occupational therapy seating interventions in Bahrain.

The top perceived barriers preventing optimal OT integration in Bahrain were lack of awareness among HCPs, limited availability of OTs, high cost of equipment, and cultural/social stigma respectively (see Figure 2).

### Association between professional characteristics, role awareness, and referral history

Pearson's Chi-Square tests revealed a highly significant association between professional discipline (Q1) and role awareness (Q4) (*Χ*^2^ = 30.33, *p* < 0.001). Specifically, certain disciplines (e.g., occupational therapy and physical therapy) demonstrated 100% awareness of the OT role, whereas others (e.g., medical doctors, nurses, social workers, and other allied healthcare professionals) showed significantly lower awareness (26.3%). However, no significant association was found between professional discipline and actual referral history (Q6) (*p* = 0.604). Similarly, work setting (Q2) did not show a significant association with role awareness (*p* = 0.934) or referral history (*p* = 0.091).

### Correlation: experience vs. Knowledge

Spearman's Rank Correlation was employed to assess the relationship between years of experience (Q3) and self-rated knowledge levels (Q5). The analysis indicated no significant correlation between these variables (rs = −0.0137, *p* = 0.887), suggesting that increased years in practice do not necessarily translate to higher self-perceived knowledge of OT services.

### Differences in perceived importance and effectiveness

**Figure 3:**
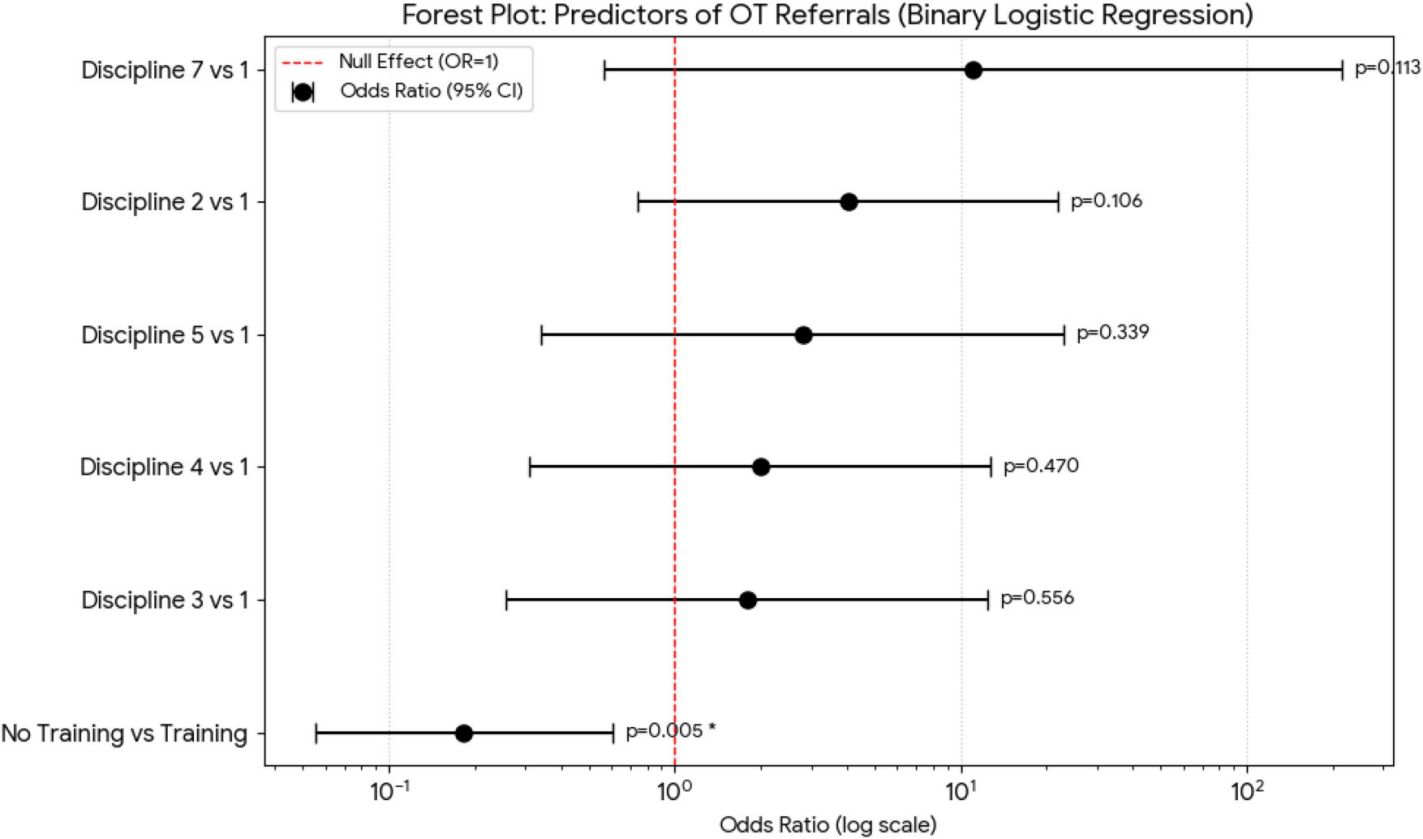
Binary logistic regression predicting occupational therapy referrals.

The Kruskal–Wallis H test revealed a statistically significant difference in the perceived importance of OT interventions across professional disciplines (H = 14.33, *p* = 0.0137). After applying the Bonferroni correction, pairwise comparisons indicated that Occupational Therapists and Physical Therapists (Median = 4.0) rated the importance significantly higher than Nurses (Median = 3.0, adjusted *p* < 0.05). The calculated effect size was 0.13, indicating a medium magnitude of difference between the groups. In contrast, no significant differences were observed across professional disciplines regarding the perceived effectiveness of OT (*p* = 0.121), suggesting a baseline level of respect for the intervention's utility across the entire multidisciplinary team. It is important to note that while these perceptions vary, they did not translate into clinical action. Our Binary Logistic Regression (see Figure 3) confirmed that formal training (*p* = 0.005), rather than professional discipline, is the only significant predictor of actual referral behavior.

### Predictors of clinical referrals

A Binary Logistic Regression was performed to identify factors influencing the likelihood of initiating a referral to OT (Q6). Binary Logistic Regression was utilized to ensure that the inclusion of specific disciplines, such as Occupational Therapy, did not skew the referral predictors. When adjusted for professional background, formal training remained the only significant predictor of referral behavior (*p* = 0.005). This indicates that the likelihood of a referral being initiated is linked to specialized education rather than the professional's specific field of practice. The overall model was statistically significant (Likelihood Ratio Test *p* = 0.032). Formal training (Q12) emerged as the only significant independent predictor of referral behavior (see Table 2). The forest plot in Figure 3 displays the Odds Ratios (OR) for each predictor. A value of 1.0 (the red dashed line) represents the “Null Effect.” The error bar for Formal Training (Q12) does not cross the null line, indicating a statistically significant impact (*p* = 0.005). The OR of 0.18 for “No Training” indicates that individuals without formal training have 82% lower odds of initiating a referral compared to those with training. While some disciplines show high point estimates for referral odds, their wide confidence intervals and *p*-values > 0.05 indicate that these differences are not statistically significant within this sample (see Figure 3). While the forest plot in Figure 3 visually summarizes the findings, Table 2 provides the complete statistical output for the Binary Logistic Regression model predicting OT referrals.

**Table 2:** Full logistic regression model for predicting OT referrals (*N* = 110).

Predictor variable	Odds ratio (OR)	95% confidence interval (CI)	*p*-value
No Formal Training (Q12)^a^	0.18	[0.05, 0.58]	0.005*
Medical Doctor (vs. PT)^b^	1.95	[0.33, 11.39]	0.556
Nurse (vs. PT)^b^	1.83	[0.30, 11.08]	0.470
Allied Health (vs. PT)^b^	2.80	[0.32, 24.16]	0.339
OT (vs. PT)^b^	4.01	[0.73, 21.90]	0.106
Other (vs. PT)^b^	10.50	[0.55, 199.30]	0.113

^a^ Reference category: “Received Formal Training”.

^b^ Reference category: “Physical Therapist”.

Model Diagnostics: No significant multicollinearity was observed between professional discipline and training; formal training remained the robust predictor (*p* = 0.005) regardless of the professional background.

## Discussion

The provided study explores how healthcare professionals (HCPs) in Bahrain perceive the role of Occupational Therapy (OT) in wheelchair seating and mobility (WSM). While OT is highly regarded, the research identifies a significant “knowledge-practice gap” where high awareness does not translate into actual clinical referrals.

There is a stark contrast between what professionals know and what they do in clinical practice. The results of this study highlight a significant discrepancy between the perceived value of occupational therapy and actual clinical practice in Bahrain. While a vast majority (80.9%) of HCPs are aware of the OT role in WSM, only 33.6% have ever referred a patient. This “knowledge–practice gap” suggests that general awareness of a profession does not automatically translate into an understanding of its specific clinical utility or the integration of its services into standard care pathways. Despite the low referral rates in the current study, HCPs recognize the theoretical value of OT. The findings in Bahrain mirror global trends where OT is often recognized as a title but poorly understood in terms of its specific scope (4, 6, 9). For instance, a 2022 study on Arab HCPs found that while 74.4% agreed that OT plays a vital role in interdisciplinary teams, 51.2% were uncertain about its main domains, and 44.8% could not distinguish it from physical therapy (PT) (10). Similarly, research in other regions has consistently shown that professions like physiotherapy and speech therapy have a better grasp of OT than physicians or nurses, a trend also observed in the current study where PTs formed the largest respondent group and showed higher awareness (11–13).

### The referral Gap and role confusion

The low referral rate (33.6%) in Bahrain is a critical finding that aligns with global literature citing “role confusion” and “overlapping” as primary barriers to interprofessional collaboration (14, 15). In many healthcare systems, WSM is viewed through a purely physical lens, leading to a bias where PTs are the primary, and often sole, recipients of seating-related referrals (16). This study's finding that 74.5% of respondents attributed the prevention of pressure ulcers to OT is a positive sign, as specialized seating for pressure management is a core OT competency. However, without formal referral protocols, these theoretical benefits remain unexploited.

### Barriers: a global perspective

The top barriers identified in Bahrain; lack of HCP awareness (80.0%), limited OT availability (76.4%), and high equipment costs (72.7%) are consistent with international scoping reviews (17). In low- and middle-income countries (LMICs), the shortage of trained OT professionals is a recurring theme; for example, Kenya reports only 0.2 OTs per 10,000 people, a workforce deficit that naturally limits referral opportunities (18). Furthermore, the high cost of equipment was a significant concern in the Bahraini cohort. This is echoed in global surveys where “services funding” and “intervention costs” are rated as the most prominent barriers to OT practice (19). The “cultural/social stigma” reported by 76.4% of Bahraini respondents is another notable barrier, which has been documented in other developing contexts where wheelchairs are sometimes viewed as symbols of “disability” rather than “enablement,” hindering patient acceptance (20–22).

The findings of this study highlight a distinct “knowledge–practice gap” among healthcare professionals regarding Occupational Therapy. While role awareness varies significantly by discipline, this awareness does not automatically translate into clinical practice (referrals). The lack of association between professional discipline and referral history (*p* = 0.604) suggests that even in professions where OT roles are well-understood, systemic or educational barriers prevent the conversion of knowledge into action (23, 24). A critical finding is the lack of correlation between years of experience and self-rated knowledge (*p* = 0.887). This contradicts the assumption that clinical exposure over time naturally increases inter-professional literacy. It underscores the need for targeted educational interventions rather than relying on “on-the-job” exposure (25). The Kruskal–Wallis results indicate that while the perceived effectiveness of OT is generally accepted across the board, the perceived importance varies by discipline. This variation suggests that some professions may view OT as an optional rather than an essential component of the multidisciplinary team, likely contributing to the observed referral gaps. These findings suggest that while the “clinical utility” (effectiveness) of OT is universally accepted, the “priority” (importance) varies by role. This variation likely stems from professional silos where nursing staff, who may have less frequent interaction with specialized seating clinics, view OT as an optional rather than essential component of the care pathway. This reinforces the study's recommendation for Interprofessional Education (IPE) to align the perceived importance of OT across all levels of the healthcare hierarchy. The logistic regression analysis identifies formal training as the most robust predictor of referral behavior. Professionals who received specific training were over twice as likely to refer patients to OT services. This suggests that the “practice” element of the gap is primarily associated with a lack of formal education on referral pathways and criteria rather than a lack of respect for the profession's effectiveness. Acknowledging the possibility of reverse causality, while formal training is a significant predictor of referral behavior (*p* = 0.005), it is possible that healthcare professionals who already actively refer patients to OT for seating are more motivated to seek out specialized training to enhance their clinical practice (26). To bridge this gap, healthcare institutions should prioritize structured inter-professional education (IPE) and formal orientation programs that define clear referral criteria for OT services (27).

### Training and interprofessional collaboration

The study highlights a critical need for structured training and better team dynamics. The critical lack of formal training (74.5%) found in this study is a widespread issue in the rehabilitation field. Evidence suggests that even when wheelchair topics are included in academic curricula, they are often not covered intensively enough to provide clinical self-efficacy (4, 28). This is corroborated by our finding that 68.2% of HCPs rated their own knowledge as low or moderate. Targeted educational interventions, such as the WHO Wheelchair Service Training Package (WSTP), have been proven to significantly increase the confidence and skills of rehabilitation assistants and therapists in diverse settings, including conflict zones (1, 29, 30). The high demand for such workshops (79.1%) in Bahrain indicates a strong readiness for professional development.

### Limitations of the current study

Despite the insights gained regarding the knowledge-practice gap, several limitations regarding the study's external validity must be noted. *Sampling and Response Bias:* The reliance on convenience sampling and digital recruitment platforms may limit the generalizability of these findings to the broader Bahraini healthcare workforce, particularly those in community or home-care settings. Additionally, despite measures to ensure anonymity, self-reported data remain susceptible to social desirability bias regarding clinical knowledge levels. *Self-Report Bias:* The data relies on self-rated knowledge and awareness, which may be subject to social desirability bias, although anonymity was maintained to mitigate this risk. *Directionality and Temporal Sequence:* As a descriptive cross-sectional survey, this study captures a single snapshot of professional perceptions and practices. Consequently, it cannot establish a definitive temporal sequence or directionality between variables. *Potential for Reverse Causation:* While formal training emerged as the most robust predictor of referral behavior (*p* = 0.005), the relationship may be bidirectional. It is possible that healthcare professionals who are already inclined to refer patients for specialized seating are more likely to seek out formal education to support their clinical decisions. *Residual Confounding*: Although the logistic regression model adjusted for professional discipline and years of experience, other unmeasured confounders may exist. Factors such as personal interest in assistive technology, specific institutional mandates, or individual patient volume could influence both training pursuit and referral patterns. *Sector Representation:* The participant pool was predominantly hospital-based (67.3%), which may lead to an under-representation of the perceptions and referral practices of HCPs working in community health, primary care, or home-care settings. *Recruitment Scope:* While the study successfully captured a multidisciplinary cohort, the reliance on digital professional networks and hospital assemblies may have excluded professionals with limited access to these platforms, potentially influencing the reported awareness levels. *Geographic Focus:* Although Salmaniya Medical Complex and other major government hospitals serve a large portion of the population, results may vary in smaller private facilities or specialized clinics not fully captured in this sample.

### Recommendations for future direction

To enhance the external validity and depth of future research, subsequent studies should move beyond the current hospital-based focus (67.3%) and digital recruitment methods to include a more representative sample of healthcare professionals from community health, primary care, and home-care settings. Future investigations should adopt qualitative or mixed-methods approaches to explore the root causes of the identified “knowledge-practice gap,” specifically investigating how cultural stigma and individual patient volume influence device acceptance and referral behavior. Furthermore, longitudinal study designs are needed to establish a definitive temporal sequence between variables, helping to clarify if formal training directly causes increased referrals or if professionals already inclined to refer are simply more motivated to seek specialized education. Researchers should also account for potential residual confounders, such as institutional mandates or personal interest in assistive technology, while expanding the geographic scope to include smaller private facilities and specialized clinics outside of major government hospitals. The researchers conclude that systemic and educational barriers currently hinder the integration of OT into WSM services in Bahrain. To improve patient outcomes, the study recommends: (1) standardized pathways implementing referral checklists within electronic medical records, (2) educational initiatives implementing of interprofessional education (IPE) workshops to bridge the awareness-knowledge gap and using tools like the WHO Wheelchair Service Training Package (WSTP) to bridge the knowledge gap, (3) policy advocacy increasing OT staffing and advocating for subsidies to reduce the high cost of seating equipment, and (4) future qualitative research studies exploring the root causes of low referral rates and the impact of cultural stigma on device acceptance.

### Strategic recommendations and implications for Bahraini healthcare

The profound “knowledge–practice gap” identified in this study where 80.9% of HCPs are aware of the OT role but only 33.6% initiate referrals suggests that awareness alone is insufficient for clinical integration. To bridge this gap, the following strategies are proposed as hypotheses for future implementation research and pilot programs within the Bahraini health system.

#### Evaluating standardized referral pathways (implementation hypothesis)

While 80.9% of professionals recognize the importance of OT, the lack of formalized pathways remains a systemic barrier. Future studies should evaluate whether integrating digital “referral checklists” into Electronic Medical Records (EMRs) increases the conversion rate of patient intake to OT seating assessments to determine if automated prompts can mitigate “role confusion” and ensure OTs are not bypassed in multidisciplinary seating decisions.

#### Pilot interprofessional education (IPE) workshops

Binary logistic regression identified formal training as the only significant predictor of referral behavior (*p* = 0.005). Implementing the WHO Wheelchair Service Training Package (WSTP) as a multidisciplinary pilot program is recommended. Structured IPE will significantly increase clinical self-efficacy and referral rates compared to the current reliance on “on-the-job” exposure, which showed no correlation with knowledge (*p* = 0.887).

#### Feasibility studies for workforce and equipment access

Systemic barriers, including limited OT availability (71.8%) and high equipment costs (63.6%), currently hinder the application of OT expertise. The Ministry of Health may consider feasibility studies on increasing OT staffing levels to test if improved availability directly reduces the “limited access” barrier reported by practitioners. Further investigation is needed into the impact of government equipment subsidies or insurance mandates on patient acceptance and device provision.

#### Addressing cultural and social stigma

With 27.3% of respondents identifying social stigma as a barrier, the effectiveness of clinical interventions may be limited by patient or societal perceptions. Future research should utilize qualitative methods to explore the root causes of stigma in Bahrain to design culturally sensitive public health campaigns that frame wheelchairs as tools for “enablement” rather than “disability”.

Supplementary Appendix B provides a SWOT analysis of the occupational therapy intervention in wheelchair seating in Bahrain as well as summarizes how these recommendations align with the study's findings, framed as exploratory opportunities rather than direct prescriptions.

## Conclusion

The findings of this study underscore a significant “knowledge-practice gap” within the Bahraini healthcare landscape. While there is a high level of general awareness (80.9%) and professional respect for the role of Occupational Therapy in wheelchair seating and mobility, this does not translate into clinical action, as evidenced by the low referral rate of only 33.6%. Healthcare professionals in Bahrain recognize the theoretical benefits of OT interventions particularly regarding postural support, independence, and pressure ulcer prevention yet they are hindered by systemic barriers, including a lack of formalized referral pathways, limited OT availability, and high equipment costs. The study further highlights a critical deficit in specialized education, with nearly 75% of practitioners lacking formal training in wheelchair seating. This educational void, coupled with the fact that only 22.7% of respondents feel interprofessional collaboration is currently adequate, suggests that professional silos remain a primary obstacle to optimal patient care. To bridge this gap, it is essential to move beyond general awareness toward targeted interprofessional education and the implementation of standardized, multidisciplinary protocols. By integrating OT-specific criteria into referral systems and addressing the high cost of assistive technology, Bahrain can better align its rehabilitation services with international standards, such as the WHO's guidelines for wheelchair provision. Ultimately, fostering a more collaborative and informed healthcare environment will ensure that wheelchair users in Bahrain receive the comprehensive, expert-led seating interventions necessary to enhance their functional independence and long-term quality of life.

## Data Availability

The raw data supporting the conclusions of this article will be made available by the authors, without undue reservation.
