# Supplementary material for: Perception of occupational therapy intervention in wheelchair seating among healthcare professionals in Bahrain
Source: Front Rehabil Sci. 2026 Apr 10;7:1797993. doi: 10.3389/fresc.2026.1797993 (PMC13106601; doi:10.3389/fresc.2026.1797993)
Supplement: Supplementary file 1 [file Supplementaryfile1.pdf]

Appendix A: Study Questionnaire

Section 1: Demographic Information

1. What is your profession?
   - [ ] Occupational Therapist
   - [ ] Physical Therapist
   - [ ] Physician
   - [ ] Nurse
   - [ ] Allied Healthcare
   - [ ] Other (please specify): ___________
2. How many years of experience do you have in your field?
   - [ ] Less than 1 year
   - [ ] 1–5 years
   - [ ] 6–10 years
   - [ ] More than 10 years
3. In which setting do you primarily work?
   - [ ] Hospital
   - [ ] Rehabilitation Center
   - [ ] Private Clinic
   - [ ] Health Center
   - [ ] Other (please specify): ___________

Section 2: Awareness of Occupational Therapy in Wheelchair Seating

4. Are you aware of the role of occupational therapy in wheelchair seating interventions?
   - [ ] Yes
   - [ ] No
5. How would you describe your level of knowledge about occupational therapy interventions in wheelchair seating?
   - [ ] Very knowledgeable
   - [ ] Somewhat knowledgeable
   - [ ] Not knowledgeable
   - [ ] Unsure
6. Have you ever referred a patient to an occupational therapist for wheelchair seating assessment or intervention?
   - [ ] Yes
   - [ ] No
   - [ ] Not applicable
7. In your opinion, how important is occupational therapy in ensuring proper wheelchair seating for patients?
   - [ ] Very important
   - [ ] Somewhat important
   - [ ] Not important
   - [ ] Unsure

Section 3: Perceptions of Occupational Therapy Interventions

8. How effective do you think occupational therapy interventions are in improving wheelchair seating for patients?
   - [ ] Very effective
   - [ ] Somewhat effective
   - [ ] Not effective
   - [ ] Unsure

9. What do you think are the main benefits of occupational therapy in wheelchair seating? (Select all that apply)
   - [ ] Improved patient comfort
   - [ ] Prevention of pressure ulcers
   - [ ] Enhanced mobility and independence
   - [ ] Better postural support
   - [ ] Other (please specify): ___________

10. What challenges do you think occupational therapists face when providing wheelchair-seating interventions? (Select all that apply)
   - [ ] Lack of resources (e.g., equipment, funding)
   - [ ] Limited awareness among healthcare professionals
   - [ ] Patient non-compliance
   - [ ] Limited training or expertise
   - [ ] Other (please specify): ___________

11. Do you think there is adequate collaboration between occupational therapists and other healthcare professionals in wheelchair seating interventions?
   - [ ] Yes
   - [ ] No
   - [ ] Unsure

Section 4: Training and Education

12. Have you received any formal training or education on wheelchair seating interventions?
   - [ ] Yes
   - [ ] No

13. If yes, did the training include the role of occupational therapy in wheelchair seating?
   - [ ] Yes
   - [ ] No
   - [ ] Not applicable

14. Do you think additional training or workshops on occupational therapy interventions in wheelchair seating would be beneficial for healthcare professionals?
   - [ ] Yes
   - [ ] No
   - [ ] Unsure

Section 5: Barriers and Recommendations

15. What do you think are the main barriers to effective occupational therapy interventions in wheelchair seating in Bahrain? (Select all that apply)
   - [ ] Lack of awareness among healthcare professionals
   - [ ] Limited availability of occupational therapists
   - [ ] High cost of equipment
   - [ ] Cultural or social stigma
   - [ ] Other (please specify): ___________

16. What recommendations do you have to improve occupational therapy interventions in wheelchair seating in Bahrain?
   (Open-ended response): ____

Section 6: Additional Comments

17. Do you have any additional comments or suggestions regarding occupational therapy interventions in wheelchair seating?
   (Open-ended response): ____
