# Supplementary material for: Perception of occupational therapy intervention in wheelchair seating among healthcare professionals in Bahrain
Source: Front Rehabil Sci. 2026 Apr 10;7:1797993. doi: 10.3389/fresc.2026.1797993 (PMC13106601; doi:10.3389/fresc.2026.1797993)
Supplement: Supplementary file 2 [file Supplementaryfile2.pdf]

Appendix B: SWOT Analysis of Occupational Therapy Intervention in Wheelchair Seating in Bahrain

STRENGTHS	WEAKNESSES
**High Professional Regard**: 71.8% of HCPs view OT involvement as "very important" and 74.5% rate it as "very effective".	**Knowledge-Practice Gap**: While 80.9% are aware of the OT role, only 33.6% have actually initiated a referral.
**Recognition of Clinical Benefits**: HCPs strongly associate OT with improved postural support (81.8%), independence (81.8%), and pressure ulcer prevention (74.5%).	**Lack of Specialized Training**: 74.5% of respondents have no formal training in wheelchair seating.
**Professional Awareness**: 100% awareness of the OT role among existing OTs and Physical Therapists.	**Poor Interprofessional Collaboration**: Only 22.7% of participants feel current collaboration levels are adequate.
**Experienced Workforce**: Over 63% of the studied healthcare professionals have more than 10 years of experience.	**Stagnant Knowledge**: No significant correlation was found between years of experience and self-rated knowledge (p = 0.887).
**OPPORTUNITIES**	**THREATS**
**Strong Demand for Training:** 79.1% of professionals expressed a desire for more workshops and professional development.	**Systemic Barriers:** Limited OT availability (71.8%) and lack of awareness among other HCPs (78.2%) act as primary roadblocks.
**Standardized Guidelines:** Implementation of the WHO Wheelchair Service Training Package (WSTP) can bridge the training void.	**High Equipment Costs:** Financial barriers were cited by 63.6% of respondents as a major hurdle to effective intervention.
**Digital Integration:** Standardizing referral pathways through Electronic Medical Records (EMRs) to prompt OT consultations.	**Cultural Stigma:** 27.3% of respondents identified social stigma regarding wheelchair use as a barrier to patient acceptance.
**Policy Advocacy:** Increasing OT staffing levels and creating government equipment subsidies to improve service delivery.	**Role Confusion:** Overlap between OT and PT roles can lead to OTs being bypassed in the referral process.

Summary of SWOT-Driven Research Directions.

Finding (Strength/Weakness)	Proposed Reform	Intended Outcome
**80.9% Awareness / 33.6% Referral**	Digital EMR Referral Checklists	Reduced Role Confusion
**74.5% Lack Formal Training**	IPE Workshops (WHO WSTP)	Increased Referral Odds
**63.6% Cite High Equipment Cost**	Subsidy Feasibility Study	Improved Device Acceptance
**71.8% Cite Limited OT Availability**	Strategic Staffing Increase	Enhanced Service Delivery
